# A Novel Aminoacyl-tRNA Synthetase Appended Domain Can Supply the Core Synthetase with Its Amino Acid Substrate

**DOI:** 10.3390/genes11111320

**Published:** 2020-11-07

**Authors:** Marc Muraski, Emil Nilsson, Benjamin Weekley, Sandhya Bharti Sharma, Rebecca W. Alexander

**Affiliations:** Department of Chemistry, Wake Forest University, Winston-Salem, NC 27109, USA; muraskmj@wfu.edu (M.M.); nilsem15@wfu.edu (E.N.); bweekley@usc.edu (B.W.); sandhya.chem2003@gmail.com (S.B.S.)

**Keywords:** tRNA, aminoacylation, aminotransferase, pyridoxal phosphate, bifunctional, channeling

## Abstract

The structural organization and functionality of aminoacyl-tRNA synthetases have been expanded through polypeptide additions to their core aminoacylation domain. We have identified a novel domain appended to the methionyl-tRNA synthetase (MetRS) of the intracellular pathogen *Mycoplasma penetrans*. Sequence analysis of this N-terminal region suggests the appended domain is an aminotransferase, which we demonstrate here. The aminotransferase domain of MpMetRS is capable of generating methionine from its α-keto acid analog, 2-keto-4-methylthiobutyrate (KMTB). The methionine thus produced can be subsequently attached to cognate tRNA^Met^ in the MpMetRS aminoacylation domain. Genomic erosion in the *Mycoplasma* species has impaired many canonical biosynthetic pathways, causing them to rely on their host for numerous metabolites. It is still unclear if this bifunctional MetRS is a key part of pathogen life cycle or is a neutral consequence of the reductive evolution experienced by *Mycoplasma* species.

## 1. Introduction

Aminoacyl-tRNA synthetases (AARSs) are multi-domain proteins that catalyze attachment of a specific amino acid to its cognate tRNA, preparing the aminoacyl-tRNAs for template-directed protein synthesis at the ribosome. AARSs have been partitioned into two classes based on evolutionarily conserved structure and sequence similarities in their catalytic domains [1,2,3]. Over time, polypeptide domains and appendages have been added to this catalytic core, increasing the complexity and functionality of the AARS family [4]. Some additions improve the overall specificity and efficiency of the AARS, for example, the editing domains found in several class I enzymes [5]. In higher eukaryotes, accretion of novel domains has endowed AARSs with functions outside of the translational pathway [6]. In other cases, the appended domain serves to regulate the multimeric state of the AARS; for example, class I methionyl-tRNA synthetase (MetRS) is either a monomer or a dimer, depending on the presence of a C-terminal appended domain [7,8]. 

As more genomic sequences become available, additional examples of idiosyncratic acquired AARS domains surface. An extreme example is that of *Mycoplasma penetrans*, an obligate intracellular human pathogen first identified in HIV-infected patients [9]. Through reductive evolution, *M. penetrans* minimized its genome to 1.36 Mb, including 1038 protein encoding genes, and thereby lost enzymes not necessary for survival in its environmental niche of the human urogenital and respiratory tracts. The MYPE9380 open reading frame (ORF) was annotated as *metS*, encoding the *M. penetrans* MetRS homolog (MpMetRS) [10]. However, *metS* contains a large insertion upstream of the canonical MetRS-encoding gene, resulting in an apparent MpMetRS protein of 1087 amino acids which is approximately twice that of a typical bacterial MetRS. BLAST analysis revealed that the far N-terminal region, consisting of 166 amino acids, does not share significant sequence similarity with any reported protein. Overall, the novel acquired domain is 46% similar to the *Anabaena* sp. alanine-glyoxylate aminotransferase (AGAT), accession number WP015216524. The *Anabaena* sp. AGAT is a member of the aspartate aminotransferase superfamily of proteins and contains a highly conserved pyridoxal 5’-phosphate (PLP) binding domain [11]. The C-terminal half of the MpMetRS ORF shares 45% sequence similarity with *Escherichia coli* MetRS (EcMetRS). A truncated version of MpMetRS encoding only the canonical MetRS core retains function and specificity similar to that of other characterized MetRSs [12,13]. 

It is of particular interest that one of the acquired domains exhibits sequence similarity with an aminotransferase, as aminotransferases are essential to the methionine salvage pathway (MSP). In this route, the enzyme would catalyze conversion of 2-keto-4-methylthiobutyrate (KMTB) to methionine, using an amino acid as the amine donor [14]. The MSP serves to salvage sulfur from 5’-methylthioadenosine, which is a by-product of polyamine (for example, spermine) and ethylene synthesis. Transaminase enzymes exhibiting activity towards KMTB are mainly aromatic transaminases or aspartate transaminase homologues [15]. Genome analysis has revealed that *M. penetrans* has lost canonical pathways for methionine biosynthesis, leaving the MSP as the only means by which it can regulate cellular methionine concentration beyond what is available from the host [16].

We sought to determine if the fusion of AGAT and MetRS genes into a single ORF arose because the AGAT is able to function cooperatively with the MetRS to supply methionine in *M. penetrans*, and, in doing so, it facilitates efficient formation of Met-tRNA^Met^. This could be accomplished by the aminotransferase domain acting upon a KMTB-tRNA^Met^ intermediate to form Met-tRNA^Met^, similar to the strategy employed in glutamine-tRNA^Gln^ formation from Glu-tRNA^Gln^ [17]. Alternatively, the transferase could act directly on KMTB to form methionine, which is then a substrate for the aminoacylation domain to synthesize Met-tRNA^Met^. Here we present a characterization of the MpMetRS aminotransferase domain, its capacity to generate methionine from the keto acid precursor, and the ability of the full MpMetRS protein to catalyze Met-tRNA^Met^ synthesis from KMTB and tRNA^Met^ substrates, using a general amine donor. 

## 2. Materials and Methods 

### 2.1. Materials

DNA oligonucleotides were synthesized by Integrated DNA Technologies (IDT; Coralville, IA, USA). HisTrap nickel columns were from Cytiva (Marlborough, MA, USA). α-^32^P ATP was purchased from Perkin-Elmer (Waltham, MA, USA). Restriction enzymes were from New England Biolabs (Ipswitch, MA, USA). Expression vector pQE70 was from Qiagen. Pfu Ultra DNA polymerase, XL-10 Gold cells and QuikChange multi-site directed mutagenesis kit were from Agilent (Santa Clara, CA, USA). SuperSignal West Pico Rabbit IgG Detection Kit was from ThermoFisher Scientific (Waltham, MA, USA). *M. penetrans* protein lysate was a gift from Dr. M. Balish of Miami University of Ohio. Salts, buffers, and growth media were from Fisher Scientific (Waltham, MA, USA).

### 2.2. Cloning, Mutagenesis, and Expression

The *metS* gene was cloned into the pQE70 vector, and the MpMetRS protein was expressed as previously described [12]. Variants Met-568→Ala, Lys-386→Ala, Asp-616→Ala and Trp-1005→Ala were generated by QuikChange mutagenesis, following the manufacturer’s protocol, with primers obtained from IDT. The M568A substitution was made to remove an internal start site within the MetRS gene and was used as the functional wild-type MpMetRS in all experiments. Three other alanine variants (K386A, D616A, and W1005A) were generated to produce catalytically inactive aminotransferase and synthetase domains, as described below. Following protein purification, all MpMetRS variants were separated by SDS-PAGE to determine homogeneity (Supplementary Materials Figure S3). 

### 2.3. Western Blot Analysis

MpMetRS samples were separated on a 10% SDS-PAGE, using 40 μg *M. penetrans* lysate, 0.1 μg recombinant MpMetRS, and 40 μg XL-Gold *E. coli* lysate containing empty vector. Proteins were transferred to a nitrocellulose membrane at 100 V for 1 h. The membrane was blocked overnight at 4 °C with 4% milk in Tris-buffered saline (pH 7.5)/0.1% Tween-20. The membrane was incubated with rabbit α-MpMetRS (ThermoScientific; 1:10,000) and rocked overnight, at 4 °C. The membrane was washed six times, incubated with goat α-rabbit-HRP (1:4000) at 4 °C for 1.5 h, and again washed six times. The remainder of the procedure was performed by the manufacturer’s guidelines for the SuperSignal West Pico Rabbit IgG Detection Kit and visualized on an Amersham Imager 600 (Cytiva, Marlborough, MA, USA).

### 2.4. In Vitro tRNA Synthesis

*M. penetrans* tRNA^Met^ (Mp-tRNA^Met^) was synthesized in vitro from a DNA template generated by extending overlapping primers (Supplementary Materials Table S2) with the Klenow fragment as previously described [18]. The duplex tDNA template (250 μL of Klenow reaction) was combined with 0.1 mg/mL bovine serum albumin (BSA), 2 mM spermidine, 250 mM HEPES·NaOH (pH 7.5), 30 mM MgCl_2_, 5 mM NTPs, 40 mM DTT, 10 U yeast inorganic pyrophosphatase, and 100 μL T7 RNA polymerase. The transcription reaction (2 mL total volume) proceeded for 12–16 h, in a shaking incubator, at 37 °C, prior to treatment with DNase I for 2 h and ethanol precipitation. The transcript was purified on a 10% polyacrylamide (19:1 acrylamide:bisacrylamide) gel with 8 M urea in 1X TBE. The tRNA band was identified by UV shadowing, excised from the gel, and eluted in 500 mM NH_4_OAc (pH 5.2) and 1 mM EDTA, overnight, in a shaking incubator, at 37 °C. The extracted tRNA was passed through a 0.2 μm syringe filter, ethanol precipitated, and resuspended in 20 mM HEPES·NaOH (pH 7.5).

### 2.5. tRNA Radiolabeling and Aminoacylation

Mp-tRNA^Met^ was ^32^P-labeled at the 3’-end, according to previously reported techniques [19]. Aminoacylation reactions were performed at 25 °C, in the presence of 20 mM HEPES (pH 7.5), 4 mM ATP (pH 7.0), 150 mM NH_4_Cl, 0.1 mM EDTA, 5 mM MgCl_2_, and 0.1 mM methionine and varying concentrations of tRNA (0.5–25 μM) with trace amounts of ^32^P-tRNA^Met^. For the two-step reaction, methionine was replaced with 10 mM 2-keto-4-(methylthio) butyrate (KMTB) and 5 mM phenylalanine with 2 μM tRNA; for alanine variants, the 2 μM tRNA was used with 100 nM MpMetRS. Reactions were quenched in 100 mM NaOAc (pH 5.2) and 0.1% SDS. Aliquots were subjected to P1 nuclease digestion and separated on cellulose TLC plates in 100 mM NH_4_OAc (pH 5.2) and 5% acetic acid following the Wolfson method [20].

### 2.6. Liquid Chromatography–Mass Spectrometry Detection of Transamination

The capacity of the MpMetRS aminotransferase domain to catalyze transamination was assayed in 100 mM ammonium bicarbonate buffer (pH 7.4), 5 mM phenylalanine, 1–40 mM KMTB, and 50 nM MpMetRS over one minute for steady-state kinetic analysis of KMTB; 100 mM ammonium bicarbonate buffer (pH 7.4), 10 mM KMTB, 0.5–16 mM phenylalanine, and 50 nM MpMetRS over one minute for steady-state kinetic analysis of phenylalanine. Screening of transamination substrates was performed, using 1 mM amino acid and 1 mM α-keto acid with 1 μM MpMetRS, over a period of 40 min. MpMetRS alanine variants were assayed at 100 nM with 1 mM KMTB and 5 mM phenylalanine with a time course of 40 min. All reactions were quenched (1:10) in Optima H_2_O/0.1% formic acid. A ZORBAX SB-C18 column (5 μm, 4.6 × 150 mm) was equilibrated for 30 min on an amaZon LC/MS with 50% Optima H_2_O/0.1% formic acid (solvent A): 50% MeOH/0.1% formic acid (solvent B) at a flow rate of 0.400 mL/min prior to sample analysis. After equilibration, a 10 μL sample was analyzed at the same flow rate, under the following LC/MS gradient: increase solvent B from 50% to 65% over 10 min, 65% to 100% over 2 min, isocratic for 5 min, 100% to 50% over 2 min, and equilibration for 10 min. 

## 3. Results

### 3.1. The MpMetRS metS Gene

The complete genome of the obligate intracellular parasite *Mycoplasma penetrans* HF-2 was sequenced in 2002 by Sasaki et al. [10]. By comparative genomics, it was clear that the annotated *M. penetrans metS* gene (3264 nt) was unusually long compared to the *E. coli* homolog (2034 nt) [12]. Sequence alignment revealed that the 3′-half of the *M. penetrans metS* gene encodes a canonical methionyl-tRNA synthetase with HIGH and KMSKS sequence motifs and 45% similarity to *E. coli* MetRS. The 5′-half of the gene is also an open reading frame with 46% similarity to the *Anabaena* sp. alanine glyoxylate aminotransferase (AGAT). We previously cloned the *M. penetrans metS* gene into a pCR2.1-TOPO vector, mutated the 12 UGA codons (used as tryptophan in mycoplasma but stop in *E. coli*) to UGG tryptophan codons, and cloned the resulting gene into a pQE70 expression vector [12]. The *Anabaena* AGAT crystal structure (PDB 1VJO) is co-crystalized with the pyridoxal 5′-phosphate (PLP) cofactor [21]. Sequence alignment of the *Anabaena* AGAT with the N-terminal domain of MpMetRS reveals that several key PLP binding residues are conserved. A spectroscopic method adapted from Wada et al. was used to determine that recombinant MpMetRS co-purifies with PLP at 72% occupancy (Supplementary Materials Table S1) [22]. Initial expression of the recombinant MpMetRS resulted in the generation of two bands on a denaturing SDS-PAGE, one at 126.4 kDa and the other at 61.4 kDa (Supplementary Materials Figure S3). The lower band was subjected to Edman sequencing analysis and corresponds to the C-terminal core synthetase domain (MpMetRSΔ). It has previously been suggested that recombinant mycoplasma genes may contain internal ribosomal binding sites, producing more than one product following heterologous expression [23]. To test whether the smaller band was due to proteolytic cleavage or an internal ribosomal binding site, we generated a Met-568→Ala variant, as Met-568 is at the beginning of the region that aligns with the canonical bacterial MetRS. The resulting overexpressed protein migrated as a single band at 126.4 kDa, suggesting that the smaller fragment was indeed the result of an internal translational start and that the M568A variant corresponds to full-length MpMetRS. 

### 3.2. Evidence for Full-Length MpMetRS In Vivo

**Figure 1:**
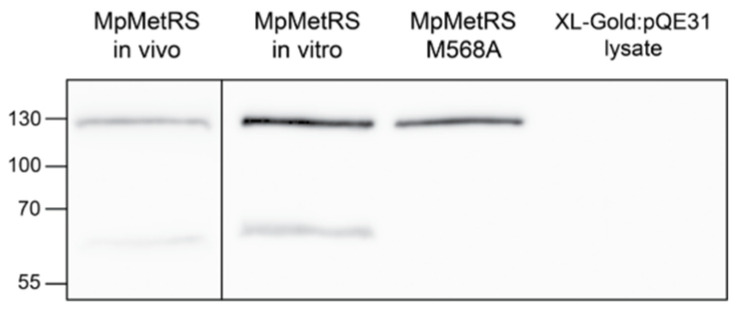
Immunoblot of in vivo and recombinant MpMetRS. Proteins were incubated with rabbit α-MpMetRS and conjugated with goat α-rabbit IgG prior to detection with Supersignal West Pico Kit. The upper band corresponds to full-length MpMetRS (126.4 kDa), while the fainter lower band is consistent with the MpMetRSΔ (61.4 kDa). The black line delineates two separate blots: one for the in vivo sample and the other for in vitro samples.

Given the presence of two products following recombinant expression in *E. coli*, we wanted to identify whether MpMetRS exists in vivo as the proposed aminotransferase-synthetase fusion protein. We therefore performed immunoblots on both *M. penetrans* cell lysate and recombinant proteins. The band present in each sample just below the 130 kDa marker is consistent with the 126.4 kDa size of full-length MpMetRS (Figure 1). An additional smaller band in the recombinant MpMetRS corresponds to MpMetRSΔ (61.4 kDa); this smaller fragment is absent in the MpMetRS M568A variant. A similarly sized smaller band is also present in the lysate sample; we hypothesize that *M. penetrans* uses an internal start codon to produce the isolate aminoacylation domain. The ratio of full-length to fragment seems variable, and ongoing experiments seek to identify the variability and significance of the alternate translational forms of MpMetRS. 

As the *Anabaena* sp. aminotransferase exists as a dimer, we probed the oligomeric state of MpMetRS. Size exclusion chromatography–multiangle light scattering (SEC–MALS) was performed to assess the molecular weight and polydispersity of the enzyme [24,25]. This analysis determined that full-length MpMetRS exists in solution as a dimer with an apparent molecular weight of 253 kDa and a polydispersity of one, while MpMetRSΔ is a monomer of 77 kDa and a polydispersity of one (Supplementary Materials Figure S1).

### 3.3. MpMetRS Is a Bifunctional Enzyme

**Figure 2:**
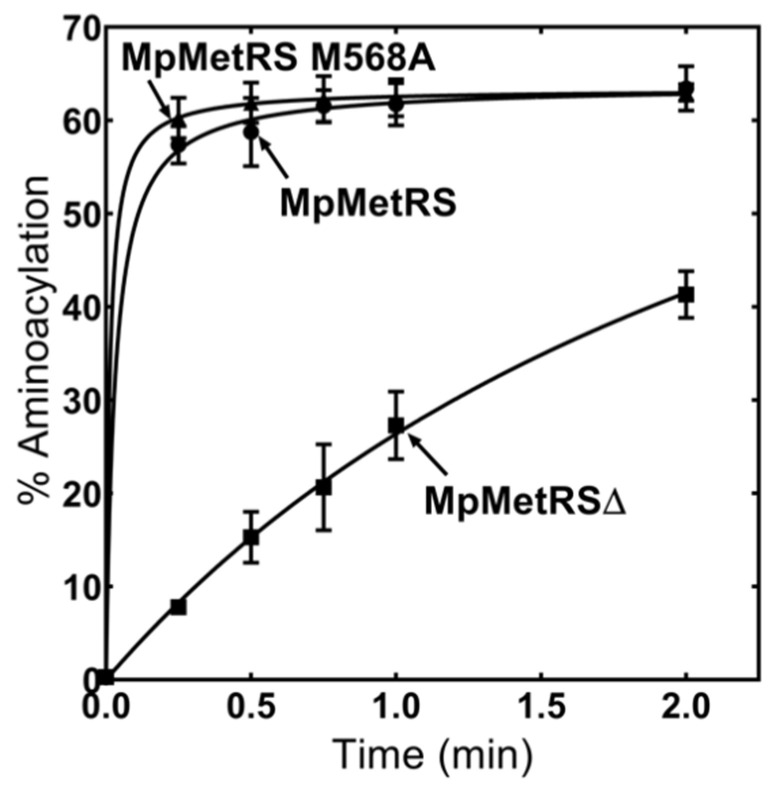
Aminoacylation of tRNA^Met^ by MpMetRS. Formation of Met-tRNA^Met^ was monitored by thin-layer chromatography of RNaseP1 fragments, using MpMetRS, MpMetRS∆, and MpMetRS M568A. All reactions used 11 μM enzyme, 0.1 mM methionine, and 0.5 μM ^32^P-labeled tRNA^Met^.

We have previously established that MpMetRSΔ alone is capable of catalyzing Met-tRNA^Met^ synthesis [12]. When the full-length MpMetRS was compared to the aminoacylation capability of MpMetRS∆, both enzymes aminoacylated tRNA^Met^; the role of the MpMetRS appended domain was unclear. Consistent with our earlier findings, aminoacylation conducted in this work, using full-length MpMetRS (M568A), remained robust for its tRNA substrate (Figure 2). While MpMetRS∆ is able to aminoacylate tRNA^Met^, the activity is low and requires an elevated enzyme concentration. Subsequent analyses of MpMetRS in this work use the M568A variant as the wild-type, full-length enzyme. 

**Figure 3:**
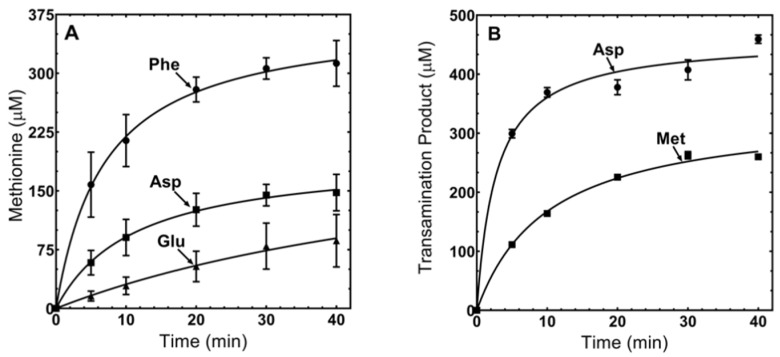
Transamination with varying amino acid and α-keto acid substrates. (**A**) Production of methionine from KMTB. Formation of methionine with varying amine donors (glutamate, aspartate, or phenylalanine) was monitored by LC–MS following reaction in the presence of 1 mM KMTB, 1 mM amine group donor, and 1 μM MpMetRS. (**B**) Production of methionine or aspartate. Synthesis of methionine or aspartate was monitored by LC-MS following reaction in the presence of 1 mM KMTB or oxaloacetate, 1 mM phenylalanine, and 1 μM MpMetRS.

The N-terminal half of MpMetRS exhibits sequence homology with class V aminotransferases such as the *Anabaena* sp. AGAT (Supplementary Materials Figure S2) [26]. We therefore set out to establish whether MpMetRS can catalyze the interconversion of α-keto acids and amino acids. The enzyme was tested with several different amine group donors and acceptors, using LC–MS to monitor reaction progress. Amine group transfer to the methionine precursor KMTB was tested with three different amino acids: phenylalanine, aspartate, and glutamate. The three amine group donors varied in their capacity as substrates, with phenylalanine being the best in vitro donor among those tested (Figure 3A).

To test the catalytic capacity of MpMetRS to use different amine group acceptors, the enzyme was supplied with either KMTB or oxaloacetate, and phenylalanine was the amine donor for production of methionine or aspartate, respectively (Figure 3B). The conversion of oxaloacetate to aspartate was quantified by measuring the depletion of phenylalanine rather than the accumulation of aspartate. In control experiments, the LC–MS signal for aspartate was not sensitive enough for accurate quantification, for reasons that remain unclear. 

### 3.4. The Domains of MpMetRS Are Functionally Independent

**Figure 4:**
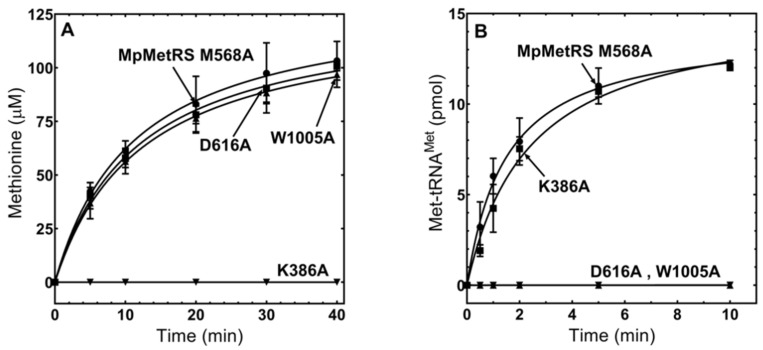
Functional independence of MpMetRS domains. Each MpMetRS variant was assayed for both aminoacylation and transamination capability. (**A**) Transamination was performed, using 100 nM enzyme with 1 mM KMTB and 5 mM phenylalanine. (**B**) Aminoacylation was performed, using 100 nM enzyme with 0.1 mM methionine and ^32^P-labeled tRNA^Met^.

While we have shown that MpMetRSΔ was capable of aminoacylation in the absence of its N-terminal domain, we wanted to determine if the two domains function independently in the context of the full-length enzyme. Site-specific substitutions in each catalytic domain were generated at essential amino acid positions. The first substitution was a Lys-386→Ala (K386A) in the aminotransferase domain; this conserved lysine aligns with the Lys-209 residue of *Anabaena variabilis* alanine-glyoxylate aminotransferase, which forms a Schiff base intermediate with PLP during amine group transfer [21]. Two individual variants were generated in the aminoacylation domain, Asp-616→Ala (D616A) and Trp-1005→Ala (W1005A). These residues align with Asp-52 and Trp-461 of *E. coli* MetRS, where the acidic aspartate orients ATP during the formation of the methionine adenylate, and the tryptophan stacks with C34 of the tRNA^Met^ anticodon [21,27,28]. To ensure there were no drastic structural change as a result of these alanine substitutions at conserved positions, we performed circular dichroism spectroscopy (Supplementary Materials Figure S4), thermal melting studies, and PLP occupancy tests (Supplementary Materials Table S1). The alanine variants were then compared to wild-type MpMetRS activity in both an LC–MS assay to monitor methionine synthesis and a TLC assay to quantify Met-tRNA^Met^ formation. For each variant, only the activity of the domain containing the substitution was inhibited, while the activity of the adjacent domain was comparable to the wild-type enzyme (Figure 4).

### 3.5. MpMetRS Aminotransferase Domain Can Supply tRNA^Met^ with Methionine

**Figure 5:**
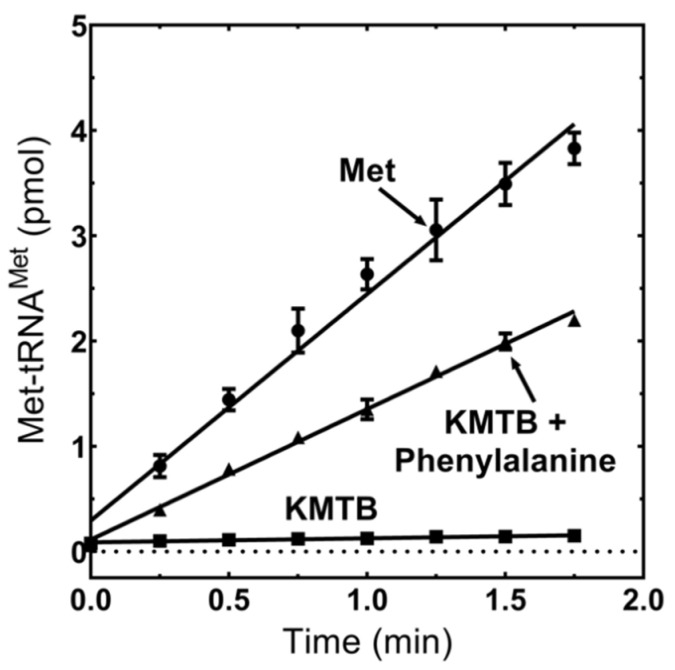
Transamination product is used as a substrate for aminoacylation. Formation of Met-tRNA^Met^ was monitored by thin-layer chromatography of RNaseP1 fragments using 0.1 mM methionine, 10 mM KMTB, 5 mM phenylalanine, 2 μM tRNA^Met^, and 50 nM *M. penetrans* MetRS. The reaction was incubated for 3 min with MpMetRS and small molecule substrates, and catalysis was initiated by the addition of Mp-tRNA^Met^.

The ability of MpMetRS to use KMTB as a transaminase substrate and the presence of fused aminotransferase and tRNA synthetase active sites led us to investigate whether the product of one domain could provide the substrate for the other. To test this hypothesis, we performed an aminoacylation assay in the presence of KMTB and phenylalanine with ^32^P-labeled tRNA^Met^. When MpMetRS is supplied with methionine directly, Met-tRNA^Met^ is formed, as previously shown. When the aminotransferase is supplied with phenylalanine and KMTB in the absence of methionine, Met-tRNA^Met^ is synthesized, albeit at reduced efficiency (Figure 5). Formation of Met-tRNA^Met^ when the enzyme is supplied Met directly is 2.2 pmol/min, which is only ~2-fold greater than the rate when Met is generated in the aminotransferase domain from KMTB and Phe (1.2 pmol/min). Aminoacylation from KMTB alone is negligible (0.04 pmol/min). The reaction kinetics of each domain were determined through either LC–MS or aminoacylation assays and are summarized in Table 1.

## 4. Discussion

While many AARSs contain appended domains that expand their functionality beyond tRNA aminoacylation [6], MpMetRS is the first known occurrence of an AARS exhibiting aminotransferase activity. This fusion protein is not observed in any of the other well-characterized members of the *Mycoplasma* spp. and is to our knowledge unique to *M. penetrans* MetRS. 

Immunoblotting of *M. penetrans* cell lysates confirmed that the full-length protein does exist in vivo. The presence of both full-length and core MpMetRS suggests that both forms are biologically relevant, although the larger polypeptide is more abundant in the cell lysates analyzed here. It remains to be seen whether the ratio of full-length to core proteins varies with cellular conditions. The likelihood that an internal ribosomal start site is responsible for the two alternate forms upon overexpression in *E. coli* is strengthened by observation of a single polypeptide product in the M568A variant corresponding to full-length protein (Figure 1).

When three different versions of wild-type MpMetRS (MpMetRS, MpMetRS∆, and M568A) were used in an aminoacylation reaction, MpMetRS and M568A aminoacylate Mp-tRNA^Met^ efficiently, while MpMetRS∆ is active but less so than the full-length enzymes (Figure 2).

Expression of the core aminoacylation domain alone (MpMetRS∆) corresponds to a structural switch from dimer to monomer. The SEC–MALS analysis for MpMetRS∆ suggests a molecular weight of 77 kDa, somewhat higher than the calculated 61.4 kDa. The MpMetRS∆ sample did display greater scatter in generated molecular weights (Supplementary Materials Figure S1), suggesting that the aminoacylation domain alone may have lower structural stability. This observation is consistent with the decreased catalytic efficiency of MpMetRS∆. While the two enzymes have similar K_M_ values for the Mp tRNA^Met^ substrate, there is a notable difference in the chemical step of aminoacylation, represented by *k*_cat_ (Table 1). While each domain can catalyze its reaction independently, as demonstrated by single substitutions at critical domain-specific positions (Figure 4), structural integrity may be necessary for full catalytic efficiency.

The aminotransferase domain of MpMetRS was shown to utilize more than one set of amine group donor and acceptor. While some aminotransferase enzymes are specific for a given amino acid-keto acid pair, others are more promiscuous in their substrate use [29,30,31,32]. Our observation that phenylalanine was the best in vitro amine group donor (Figure 3A) was consistent with work of Berger and coworkers, who noted that aromatic and branched chain amino acids are often amine donors in methionine regeneration [33,34]. It was surprising to note that oxaloacetate was a better keto acid substrate for the aminotransferase activity (Figure 3B); the optimal in vivo substrate remains unknown. It may be that the MpMetRS transaminase domain has been co-opted to catalyze Met synthesis in the context of the fused enzyme (keeping in mind the lack of substrate stringency for many aminotransferases) but has a primary function elsewhere as an aspartate transaminase.

We have shown here that the fused aminotransferase domain is capable of producing methionine that can be used by the core synthetase domain to synthesize Met-tRNA^Met^. Over the course of two minutes, the initial rate for Met-tRNA^Met^ synthesis beginning with KMTB and Phe was half that observed with methionine added directly to the reaction (Figure 5). For the net aminoacylation reaction, phenylalanine and KMTB were preincubated with enzyme for three minutes prior to tRNA addition to allow for methionine accumulation. Without this pre-incubation, there was a 15 s lag in product formation, although the rate following the lag period was not significantly different from the linear initial rate shown here.

Given the two distinct active sites on this novel enzyme, and the capacity for the synthetase domain to use methionine produced by the aminotransferase domain, the question of methionine transport arises. Crystal structures exist for isolated proteins homologous to these domains: *E. coli* MetRS (PDB 1QQT) and *Anabaena* sp. AGAT (PDB 1VJO) [21,34]. However, the orientation of the domains relative to one another and the arrangement of the monomers within the dimer are both unknown. We have considered whether methionine could migrate through substrate channeling in MpMetRS. Substrate channeling between distal enzyme active sites is best exemplified by amidotransferases such as glutamine phosphoribosylpyrophosphate (PRPP) amidotransferase (GPAT). This enzyme hydrolyzes the glutamine side chain amide and shields the NH_3_ group from aqueous environment through a hydrophobic tunnel to the PRPP active site 16 Å away [35]. A rationale for the presence of substrate channeling is either to protect a labile substrate from hydrolysis or to prevent its use by other enzymes [36]. One evolutionary advantage to the fused MpMetRS could be the direct delivery of methionine for attachment to tRNA^Met^, while preventing enzymes like S-adenosylmethionine synthase from competing for its common substrate. The MpMetRS fusion protein could allow direct transfer of methionine from the aminotransferase domain to the aminoacylation domain, either within one monomer (intramolecular channeling) or across the dimer interface (intermolecular channeling). Alternatively, methionine may simply be released from the aminotransferase active site for use by a neighboring enzyme. While the existence of a bifunctional enzyme does not necessitate substrate channeling, there is precedent for monofunctional enzymes that exist as a fusion protein in another organism. One clear example is the thymidylate synthase (TS) and dihydrofolate reductase (DHFR) enzyme pair; in humans, they are isolated monofunctional enzymes, while in some protozoan species, they are bifunctional fusion proteins [37]. Substrate channeling has been demonstrated for some of the protozoan bifunctional TS-DHFR proteins. While we do not know if MpMetRS exhibits channeling, it has been suggested that synchronized reaction rates would facilitate channeling [38]. Our kinetic studies show that the reaction rates in the two domains are similar (*k*_cat_ ~6 s^−1^). Additionally, there was little benefit to providing the enzyme time to produce methionine prior to tRNA addition, suggesting that simple diffusion may not explain how the MetRS sequesters its methionine substrate. Future work includes structural investigations of the individual catalytic domains and the full protein fusion to understand whether substrate channeling is possible.

The question remains as to why *M. penetrans* would retain such a gene fusion in the context of a condensed genome lacking many key metabolic enzymes [39,40]. As these bacteria are obligate intracellular parasites, they obtain most molecular building blocks from their host rather than through canonical biosynthetic pathways; indeed, *M. penetrans* lacks methionine biosynthesis enzymes. Use of an aminotransferase would enable *M. penetrans* to increase the local concentration of methionine, using KMTB and an amine-donating amino acid. Synthesis of methionine from KMTB is the last step in the methionine salvage pathway; our data suggest that the N-terminal domain of MpMetRS is a transaminase for methionine production.

Recombination events during genomic erosion of mycoplasma species may have led to unique protein fusions like the one seen with MpMetRS. The essentiality of the MpMetRS appended domain remains unknown, despite the demonstration here that it possesses aminotransferase activity in vitro. Until recently, the extreme N-terminal 166 amino acids of MpMetRS did not align with any known proteins; however, a PHYRE2 structure-based search of the MpMetRS appended domain suggested homology to another fusion protein, phosphonate-specific cytidylyltransferase, and 2-aminoethylphosphonate transaminase (PDB 6PD1), from the bacterium *Treponema denticola* [41]. The 2-aminoethylphosphanate aminotransferase (AEPT) is characterized as a Class V aminotransferase, consistent with our earlier BLAST results aligning MpMetRS with the *Anabaena* spp. AGAT. It remains to be determined if the MpMetRS aminotransferase has an affinity for 2-aminoethylphosphonates or whether it possesses nucleotidyltransferase capabilities suggested by this new alignment. Future work will focus on structure determination of this novel fusion protein and its possible role in the metabolic life cycle of the human pathogen *M. penetrans*.

## 5. Conclusions

In conclusion, the results presented here demonstrate the first example of an AARS for which the canonical aminoacylation activity is augmented by aminotransferase function. The capacity for MpMetRS to generate its own methionine substrate from a metabolic precursor suggests an intriguing adaptation to environmental conditions. It remains to be determined whether this catalytic duality contributes to the *M. penetrans* life cycle and whether the acquisition of the aminotransferase domain is unique among AARS enzymes or may yet be identified in additional species.

## Figures and Tables

**Table 1:** MpMetRS kinetic parameters.

AARS Domain Kinetics	Substrate	K_M_ (μM)	*k*_cat_ (s^−1^)	*k*_cat_/K_M_ (M^−1^ s^−1^)
	M568A:tRNA^Met^	2.7 ± 0.7	6.2 ± 1.5	2.30 × 10^−6^
	MpMetRS∆:tRNA^Met^	2.3	0.5	0.22 × 10^−6^
ADT Domain Kinetics	Substrate	K_M_ (mM)	*k*_cat_ (s^−1^)	*k*_cat_/K_M_ (M^−1^ s^−1^)
	KMTB	4.1 ± 0.3	6.9 ± 0.7	1.7 × 10^−3^
	Phenylalanine	0.5 ± 0.1	5.4 ± 0.4	10.9 × 10^−3^

Note: MpMetRS∆ kinetic parameters were previously reported by Jones et al. [12].

## Supplementary Materials

The following are available online at https://www.mdpi.com/2073-4425/11/11/1320/s1. Figure S1: SEC–MALS of MpMetRS. Figure S2: Sequence alignment of MpMetRS and other Class V aminotransferases. Figure S3: SDS-PAGE of purified MpMetRSs, Figure S4: Circular dichroism of MpMetRS alanine variants. Table S1: MpMetRS structural properties. Table S2: MpMetRS tRNA^Met^ in vitro primers.
