# Supplementary material for: A Novel Aminoacyl-tRNA Synthetase Appended Domain Can Supply the Core Synthetase with Its Amino Acid Substrate
Source: Genes (Basel). 2020 Nov 7;11(11):1320. doi: 10.3390/genes11111320 (PMC7694997; doi:10.3390/genes11111320)
Supplement: Supplementary file 1 [file genes-11-01320-s001.pdf]

**Supplementary Data**

SEC-MALS

The approximate molecular weight of MpMetRS was determined using size exclusion chromatography coupled with multiangled light scattering [23,24]. The recombinant MpMetRS M568A [1 mg/mL] or MpΔMetRS [2 mg/mL] was equilibrated in SEC-MALS buffer (50 mM Sodium Phosphate•NaOH (pH 8.0), 250 mM NaCl and 5% glycerol). The protein was resolved using an analytical size exclusion TSKgel column (7.8 mm × 30 cm, 8 µm particle size; Tosoh Bioscience) at a flow rate of 0.5 mL/min over 35 minutes. Elution was monitored by $UV_{280}$ absorbance and molecular mass determined by MALS (HELIOS II; Wyatt Technology) with linked refractive index determined (Oprilab rEX; Wyatt Technology). Astra 6.1 software (Wyatt Technology) was used to analyze peaks based on $UV_{280}$ and figures were generated using Prism Graphpad 8.0.

PLP Occupancy

Extraction of pyridoxal 5'-phosphate PLP was adapted from Wada, L. et al [21]. A PLP stock solution was made using 20 mg PLP dissolved in 10 mM Tris, pH 8.0 in a 100 mL volumetric flask. A range of standards was generated using the PLP stock (0-75 nmol) in 10 mM Tris, pH 8.0 in 600 µL. The MpMetRS sample (50 nmol) was diluted in 10 mM Tris, pH 8.0 in 600 µL used directly after purification and dialysis. To each sample, 70 µL of 5 M NaOH was added and the reaction was placed at 70 °C for 10 minutes. To each sample 35 µL HCl [12 N] and 150 µL Tris, pH 8.0 [1 M] and centrifuge for 5 minutes at 12k rpm. The resulting supernatants were placed into clear 96 well plates and their absorbance measured at 415 nm wavelength.

Circular Dichroism and Thermal Stability

The MpMetRS samples were dialyzed twice in 1X PBS buffer (pH7.5) and the $A_{280}$ was monitored on a Cary 50 UV-Vis spectrophotometer to asses protein concentration. In a 0.1 mm quartz cuvette the 0.1 mg/mL protein samples were run at 10 nm/min from 200-260 nm with three scans at 25 °C. Samples were then tested for their thermal stability by increasing the temperature 2 C/min from 20-80 °C. Samples were analyzed using a Jasco J-720 spectropolarimeter.

Supplemental figures

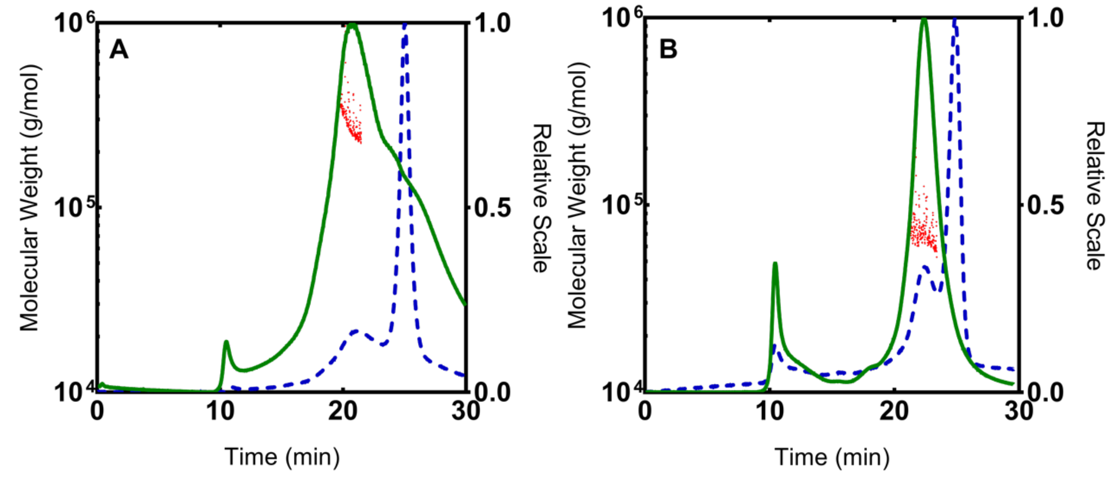

**Figure S1. SEC-MALS of MpMetRS.** A 1 mg/ml MpMetRS or 2 mg/mL MpΔMetRS sample was passed through a HPLC system and filtered through a Wyatt Dawn Helios-II and Optilab rEX to measure the light scattering and refractive index of the protein. The absorbance at 280 nm was monitored by a Waters 2417 absorbance detector. SEC-MALS showed the molecular weight at the peak reading was 253 kDa and monodisperse indicating the dimeric state of the MpMetRS protein and 77 kDa and monodisperse for MpΔMetRS. The $A_{280}$ trace is represented as a solid green line, the differential refractive index (dRI) is represented by a blue dashed line and the molecular weights are represented by red dots. Molecular weights are plotted on the left y-axis while the UV and dRI trace were normalized to 1 and plotted on the right y-axis.

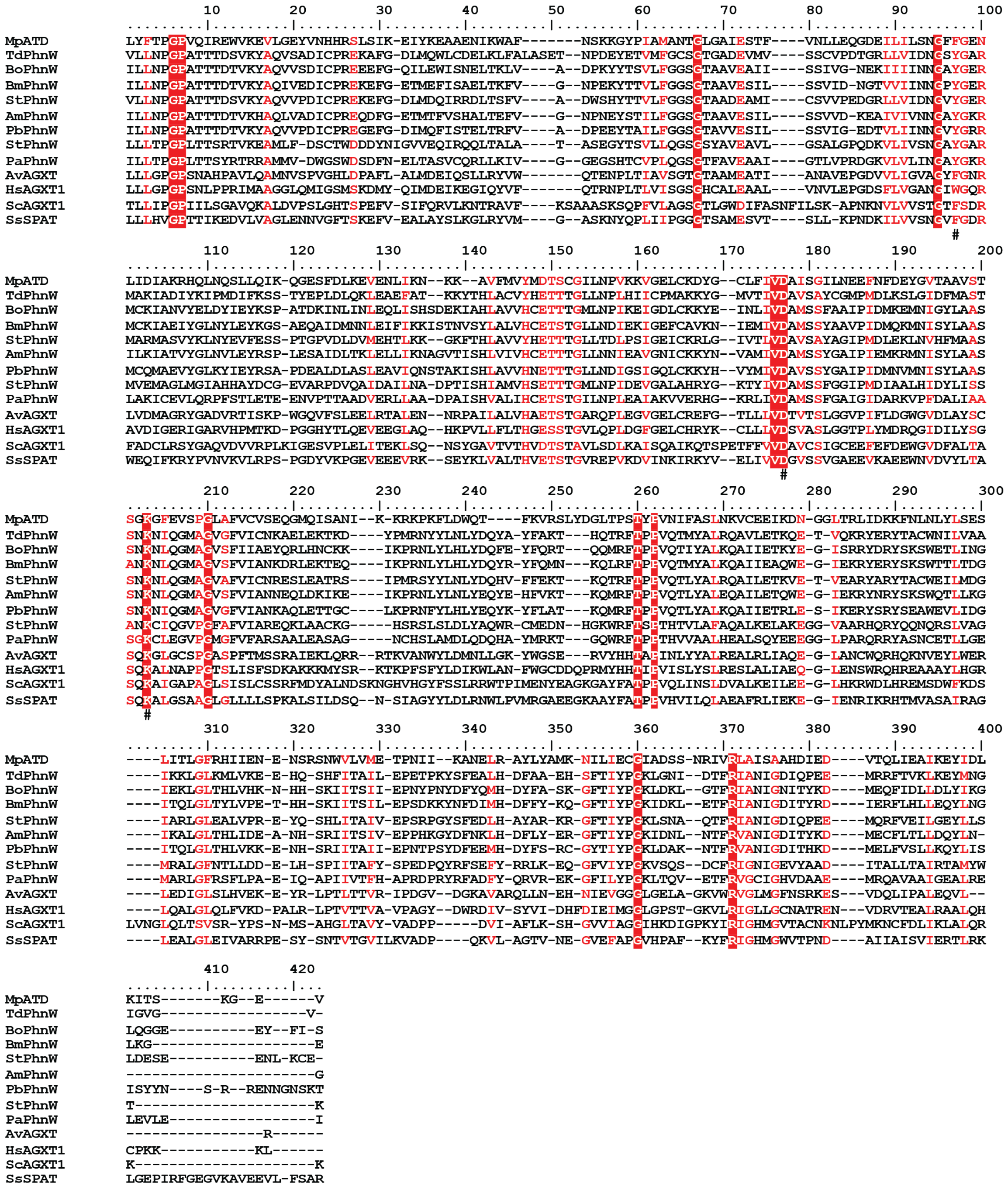

**Figure S2. Sequence alignment of MpMetRS and other Class V aminotransferases.** Aminotransferase domain (ATD), 2-Aminoethylphosphonate-pyruvate aminotransferase (AEPT), Alanine-glyoxalate aminotransferase (AGXT), Serine-pyruvate aminotransferase (SPAT); *M. penetrans* ATD, *T. denticola* AEPT, *B. obstructivus* AEPT, *B. megaterium* AEPT, *S. thermophilla* AEPT, *A. macyae* AEPT, *P. bacterium* AEPT, *S. typhimurium* AEPT, *P. aeruginosa* AEPT, *A. variabilis* AGXT, *H. sapiens* AGXT1,

*S. cerevisiae* AGXT, *S. solfataricus* SPAT; Catalytically important residues are denoted by #. All sequences were truncated to the first region of homology due to several being fusion proteins. Multiple sequence alignment was generated using T-coffee MSA service [25].

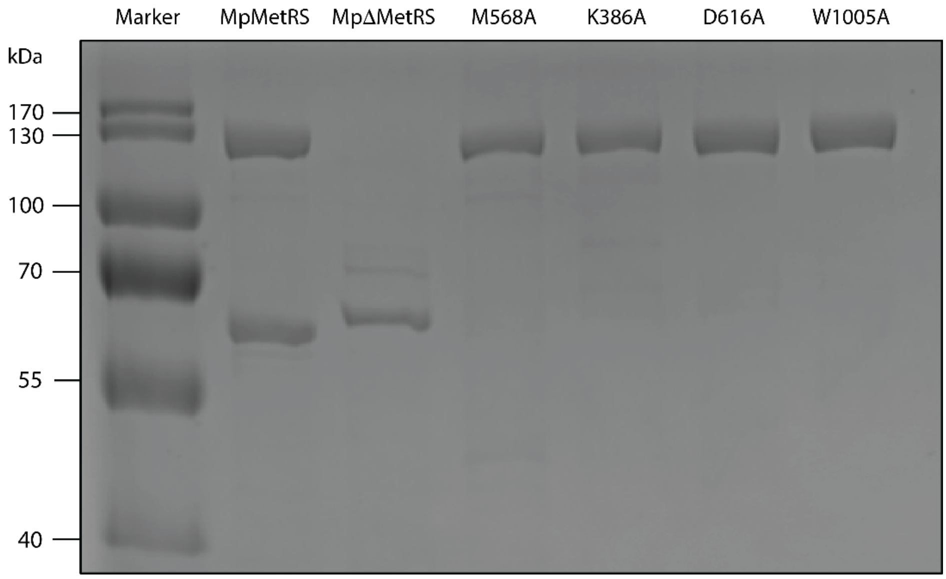


**Figure S3. SDS-PAGE of purified MpMetRSs.** MpMetRS samples were separated on a 10% SDS-PAGE followed by Coomassie Blue staining. The expected size of full-length MpMetRS is 126.4 kDa and 61.4 kDa for MpΔMetRS.

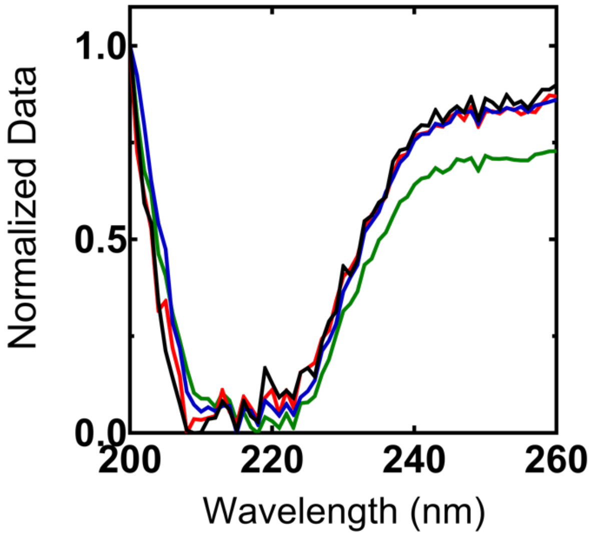


**Figure S4. Circular dichroism of MpMetRS alanine variants.** Circular dichroism spectroscopy was performed in 1X PBS (pH 7.5) scanning from 200-260 nm at 25 °C; M568A is in black, K386A in blue, D616A in red and W1005A in green.

**Table S1. MpMetRS structural properties.**

| | M568A | K386A | D616A | W1005A |
|---|---|---|---|---|
| PLP Occupancy | 72 ± 9.1 % | 66.2 ± 11.2 % | 63.5 ± 6.6 % | 63.8 ± 6.9 % |
| Thermal Stability | 54.0 | 55.7 | 52.3 | 57.8 |

**Table S2. MpMetRS tRNA$^{Met}$ *in vitro* primers.**

| | Forward Primer | Reverse Primer |
|---|---|---|
| Mp-tRNA$^{Met}$ | AATTCCTGCAG**TAATACGACTCACTATA**GGCAGAGTATCTCAGTGGTTAGAGAACTCGGCTCATACCCGAGG | mUmGGTGACAGAGGAGAGATTCGAACTCTCGACACCTCGGGTATGAGCCGAG |

*T7 promoter in bold and overlapping regions are underlined.
